# Highly sensitive and selective antibody microarrays based on a Cy5-antibody complexes coupling ES-biochip for *E. coli* and *Salmonella* detection †

**DOI:** 10.1039/d2ra03391g

**Published:** 2022-08-31

**Authors:** Timpika Hormsombut, Patsamon Rijiravanich, Werasak Surareungchai, Surachate Kalasin

**Affiliations:** Faculty of Science and Nanoscience & Nanotechnology Graduate Program, King Mongkut's University of Technology Thonburi Bangkok 10140 Thailand surachate.kal@kmutt.ac.th; BioSciences and Systems Biology Research Team, National Center for Genetic Engineering and Biotechnology, National Sciences and Technology Development Agency at King Mongkut's University of Technology Thonburi Bangkok 10150 Thailand patsamon.rij@biotec.or.th; School of Bioresources and Technology, King Mongkut's University of Technology Thonburi Bangkok 10150 Thailand

## Abstract

Foodborne pathogens are threats in food and a cause of major health issues globally. Microbial safety has become a key concern to eliminate disease-causing pathogens from the food supply. For this purpose, the Cy5 dye conjugated with a double-biotin DNA linkage and a detection antibody (Cy5-Ab complexes) was developed to amplify a foodborne detection signal on a microarray. Additionally, the ES-biochip was designed to attain a visual screening of an antibody microarray for the simultaneous threat detection of *Salmonella* and *Escherichia coli* (*E. coli*). Quantification was also performed by fluorescence. After optimizing the Cy5-Ab complex appendage and enhancing the detection signal from a sandwich immunoassay, high sensitivity and selectivity were observed. The limits of detection for both pathogens in buffer and food samples were 10^3^ CFU mL^−1^ and less than 9 CFU mL^−1^ by visual screening and fluorescent intensity quantification, respectively. Mono and duplex responses were not significantly different which means that no cross-reactivity occurred. Uniquely, the assays hold great potential to be used in several fields, such as clinical diagnosis of foodborne microbes, food hygiene screening, and pathogen detection.

## Abstract 2

A visual ES-biochip was highly sensitive and selective as well as enabled simultaneous detection. An optimized amount of Cy5 dye was attached to a Cy5-Ab complex label using a double-biotin DNA linkage.

## Graphical abstract

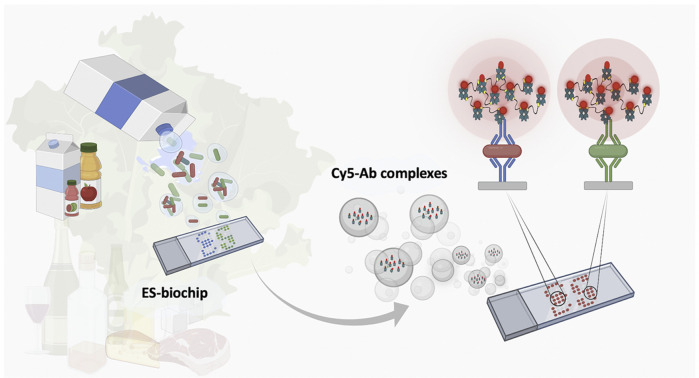

## Introduction

Foodborne pathogens are microorganisms contaminating food or water that can cause a foodborne disease.^1^ Several reported foodborne microorganisms are found in freshly produced and commercial products with pathogenic bacteria, viruses, and parasites. Among these, bacteria are the most common group of pathogens that cause foodborne illnesses.^2^ Foodborne pathogen detection is of major importance in the food sector, as food production management is required to maintain consumer safety. For example, the toxin produced by *E. coli*, which causes diarrhea or kidney failure in patients, can result in life-threatening consequences.^3^ According to the Centers for Disease Control and Prevention (CDC), as of 2011, *Salmonella* is one of the most common foodborne pathogenic bacteria, with up to 4000 cases reported annually in the United States (USA).^4^ FoodNet reported that the pathogenic bacteria caused 25 606 infected cases and 120 deaths per 100 000 individuals in 2018. Referring to this report, *Salmonella* was the most common bacterial infection with 9084 cases and 36 deaths followed by Shiga toxin-producing *Escherichia coli* (STEC) infection with 2925 cases and 13 deaths. During the years 2015–17, there was a significant increase of 9% for *Salmonella* and 26% for STEC in the number of bacterial infections diagnosed by culture-independent diagnostic tests (CIDT).^5^ As mentioned above, the data shows that controlling these bacteria and their early detection in food is very important and beneficial to the food industry.

Regularly, the gold standard for the detection of foodborne pathogens is traditional methods for the detection of foodborne pathogens, including microbiological methods, polymerase chain reaction (PCR), and enzyme-linked immunosorbent assay (ELISA). In particular, cell plate culturing is a very time-consuming technique that normally takes 2–7 days to confirm contamination of foodborne pathogens.^6^ The PCR approach, on the other hand, uses DNA analysis as a molecular diagnosis.^7^ Nevertheless, this method involves a rather cumbersome sample preparation process to obtain DNA for analysis and requires skilled personnel.^8^ Meanwhile, ELISA has been used as a diagnostic tool in biotechnology, as well as a quality control check in various foodborne pathogens that relies on the specificity of antibody–antigen interaction.^9^ The recent development of immunological-based methods in several studies has made it easier and faster to detect pathogens in food, such as lateral flow immunoassay (LFA). LFA is a paper-based method for detecting and quantifying an examined target in a complex mixture by placing a sample on a test device and displaying it, resulting in widespread use due to low production costs and ease of use.^10^ However, a major drawback of the LFA methods is the obstruction of pores due to matrix components and the need for repeated trials to confirm the results of the analysis, as the test may require more than one confirmation, whereas LFA can only be measured one at a time.

When compared to the approaches discusses above, microarray-based assays improved high throughput, high reproducibility for one measurement only, quantitative pathogenic detection, and allowing for mass production of antibody microarrays with a use of microdroplet technology.^11^ As noted, there are different types of microarrays, such as DNA microarrays, antibody microarrays, cellular microarrays, protein microarrays, etc. Various platforms for microarrays are now used for microbial diagnostics where microarrays can be classified according to specific characteristics such as probe characteristics or the specific method used for probe positioning for target detection.^12^ The original DNA microarrays were initially used for gene expression analysis. Recently, a DNA microarray has been used to screen for multiple pathogens isolated in different diagnostic kits.^11^ Most pattern arrays commonly use rows or columns to indicate the position of targets or the number of replications, such as pattern of *E. coli* O157:H7 and *Salmonella* spp. on nitrocellulose membrane and poly-l-lysine (PLL) glass slide.^13^ Bian *et al.*, 2020 designed a pattern array in the form of letters to ease an observation with fluorescent spots.^14^ Most microarray detection techniques use fluorescent labeling, which is bonded to the target molecule, allowing the analysis of single or multiple color effects in the same microarray.^15^ Moreover, fluorescence immunoassay can be used for the detection of several pathogenic targets. It's a sensitive technique including antibodies and in the quantification of antigens such as virus or bacteria.^16^ Goswami *et al.*, 2004 using Cy3=Cy5-bearing alkylation agents for DNA labeling. The advantage of this method is that it can be labeled at any step of the hybridization.^17^ In 1983, antibody microarrays (antibody matrix) were first introduced by Tse Wen Chang in a scientific publication.^18^ After that, the system has been continuously developed and integrated with the detection of pathogens in food. Gehring *et al.*, 2008 developed a 96-well microplate base on antibody microarray detect *E. coli* O157:H7 and *S. typhimurium* by using Cy3-labeled reporter antibodies with sandwich assay with assay time of 2.5 h.^19^ Later, Charlermroj *et al.*, 2011 used foodborne antibody array (FAbA) chip compared with ELISA (conventional method) by using chemiluminescent for *E. coli* O157:H7 and *Salmonella* spp. detection. In comparison with about 4 h for ELISA while FAbA takes 1.15 h. Both methods give the same sensitivity of 8 × 10^4^ CFU mL^−1^ of *E. coli* O157:H7 and 5 × 10^7^ CFU mL^−1^ of *Salmonella*.^20^ However, despite some interesting features, most microarrays have integrated detection systems that need to be developed and require further improvements in sensitivity and stability.

The scope of this work was to exploit this antibody microarray technology for the duplex detection of *Salmonella* and *E. coli* using *Salmonella typhimurium* (*S. typhimurium*) and *E. coli* O157:H7 as models. The biochip array of *E. coli* and *Salmonella* (ES-biochip) was designed to make observing measurement results easier by using liquid-droplet technology, much low amount of capture antibody used. Conferring a design pattern of the fluorescent immunoassay, it relied on the detection signal amplified by including the double-biotin DNA linkage bind with Cy5-streptavidin and detection antibody (Cy5-Ab complexes) were used for improvement of sensitivity and to reduce the detection steps for sandwich immunoassay. Ultimately, this antibody sensor was demonstrated by quantitatively detecting pathogens contaminating milk and mixed fruit juice. With this outcome, the developed laboratory-based platform microarray allowed simultaneous detection of foodborne pathogens with excellent accuracy while reducing assay time, paving the way for portable detection sensors in a resource-limited area with rapid detection.

## Materials and methods

### Apparatus

Antibodies arrays were fabricated using a BioSpot BT600 GmbH (Germany) with BioSpot control software. The working slide was a SuperFrost® Plus from Thermo Fisher Scientific GmbH (Germany). A fluorescence image was performed by using Biotek Cytation 5 from BioTek® Instruments GmbH (Germany) with Gen5 3.05 Imager program.

### Materials

All chemicals used in this work were analytical grade and were used as received. EDTA and tween-20 were purchased from Sigma-Aldrich. Bovine serum albumin, fraction V, (BSA) was from Merck. The Cy5-streptavidin was purchased from Abcam US (United States). Phosphate buffer saline (1× PBS, 1.8 mM Na_2_HPO_4_·7H_2_O, 10 mM KH_2_PO_4_, 137 mM NaCl, and 2.7 mM KCl, pH 7.4) was used for all solution preparations. 1× PBS was used as working buffer, PBS containing 0.05% tween-20 was used as a washing buffer, and PBS containing 0.05% tween-20 and 2% (w/v) BSA was used as a blocking buffer. All solutions and buffers were prepared using sterile Type 1 pure water from Merck Millipore.

The double-biotin DNA linkage, 24 base poly-T oligonucleotide with the 3′ and 5′ biotin-modified DNA, was purchased from Integrated DNA Technologies (USA). Goat anti-*E. coli* O157:H7 (#5310-0326), biotin-labelled goat anti-*E. coli* O157:H7 (#16-95-90), goat anti-*Salmonella* CSA-1 (#5310-0322), and biotin-labelled goat anti-*Salmonella* CSA-1 (#5360-0031) were purchased from KPL BacTrace® US (United States).

### Preparation of bacteria cell

Stock cultures of *E. coli* O157:H7 (DMST 4212), *S. typhimurium* (ATCC 14028) and *Listeria monocytogenes* (DMST 3180) were obtained from the Department of Medical Sciences Thailand and American Type Culture Collection (Manassas, VA).

Cultures were prepared by incubating the bacteria in nutrient broth (HiMedia) at 37 °C for 18 h, then separated into two portions for determining the cell numbers and using in the assay. To determine cell numbers, serial 10-fold dilutions of cultured cells were immediately made in 0.9% saline solution, and then 0.1 mL of proper dilutions were surface plated onto nutrient agar (HiMedia). After incubation at 37 °C for 24 h, the colonies of each bacterium on the plates were counted to determine the colony forming units per milliliter (CFU mL^−1^). To prepare cells for use in the assay, 1 mL of cultured cells was immediately centrifuged at 8000 rpm for 10 min. The supernatant was discarded and resuspended the cell pellet in 1 mL of sterile Type 2 water. This centrifugation/resuspension cycle was repeated three times and the cells were then resuspended in 1 mL working buffer and stored at 4 °C, until known the cell numbers. The refrigerated bacteria solution was diluted with a working buffer to the required concentration. Finally, cells were heat-killed by placing 1 mL of each known concentration and 10 μL of 1 mM EDTA (pH 7.4) in a 1.5 mL tube and boiling in a water bath for 20 min for safety considerations of testing^13,19,21^ and then kept at 4 °C until used.

### Preparation of Cy5-Ab complexes

A 100 μL of 60 μg mL^−1^ Cy5-streptavidin was mixed with 100 μL of 60 fM double-biotin DNA linkage and incubated for 30 min at room temperature. After that, 100 μL of 20 μg mL^−1^ of the detection antibody (biotin-labelled goat anti-*E. coli* O157:H7 or biotin-labelled goat anti-*Salmonella* CSA-1) were added to the mixed solution and subsequently incubated for 30 min in room temperature. The reporters Cy5-Ab complexes were received and kept at 4 °C until use. For duplex detection on ES-biochip, anti-*E. coli*/Cy5-complexes and anti-*Salmonella*/Cy5-complexes were immediately mixed at a 1 : 1 volume ratio before being used.

### Silver staining DNA for TEM measurement

A 5 μL aliquot of Cy5-double biotin DNA linkage and Cy5-Ab complexes were dropped on a carbon grid for 30 min at RT and blotted with filter paper, leaving a small amount of the solution on the carbon grid surface. Then, a 5 μL ammoniacal Ag solution of 10% NH_4_OH with 0.1 mM AgNO_3_ was added and blotted with a filter paper for 10 s. The prior reaction was stopped with 5 μL of formaldehyde reduction from 10% NH_4_OH with 10% CH_2_O mixed solution for 30 min, then blotted with the filter paper and dried with N_2_ gas. The specimens were examined with a Hitachi HT7700 electron microscope (Japan), operated at an accelerating voltage of 100 kV.

### Preparation of antibody arrays for ES-biochip

The ES-biochip with an array of 11 × 9 = 99 spots was designed to facilitate visual observation of E (for *E. coli*) and S (for *Salmonella*). The negative (NC) and positive (PC) control spots were integrated into the same array. Each ES-bioship array is composed of 10 spots of E, 10 spots of S, 51 spots of NC and 28 spots of PC, as shown in Scheme 2A. PipeJet Pipe 200-S (200 μm of inner diameter) was used for all spots. The desired single droplet volumes with 5% tolerance were calibrated before injecting a droplet on the slide. The advanced dispenser control was set up as 83% PipeJet stroke, 112 μm ms^−1^ of PipeJet stroke velocity, by 1 shot at 1 Hz of frequency with 0.059 s of dispense time. The 99 batches of control were set up using 100 s of stroke and 70 μm ms^−1^ of stroke velocity by one shot at 1 Hz of the frequency. Ten and fifteen nanoliters of droplets were generated and spotted with 1 mm gap for each one, and *X*–*Y* axis speed was 1 mm s^−1^. Ten and fifteen nanoliters of capture antibodies (prepared in 1× PBS, pH 7.4) were continuously spotted on SuperFrost® Plus slide. The spotting solution experiments; 150 μg mL^−1^ of anti-*E. coli* O157:H7 and anti-*Salmonella*, 20 μg mL^−1^ IgG-biotin and 0 μg antibody were used for E, S, PC and NC, respectively. The antibody array was then incubated in a silica box overnight. After that, a 150 μL of blocking buffer were incubated on the slide at room temperature (RT) for 1 h. After rinsing 3 times with washing buffer for 5 min each, the ES-biochip arrays were immediately tested or stored at 4 °C until use.

**Scheme 1:**
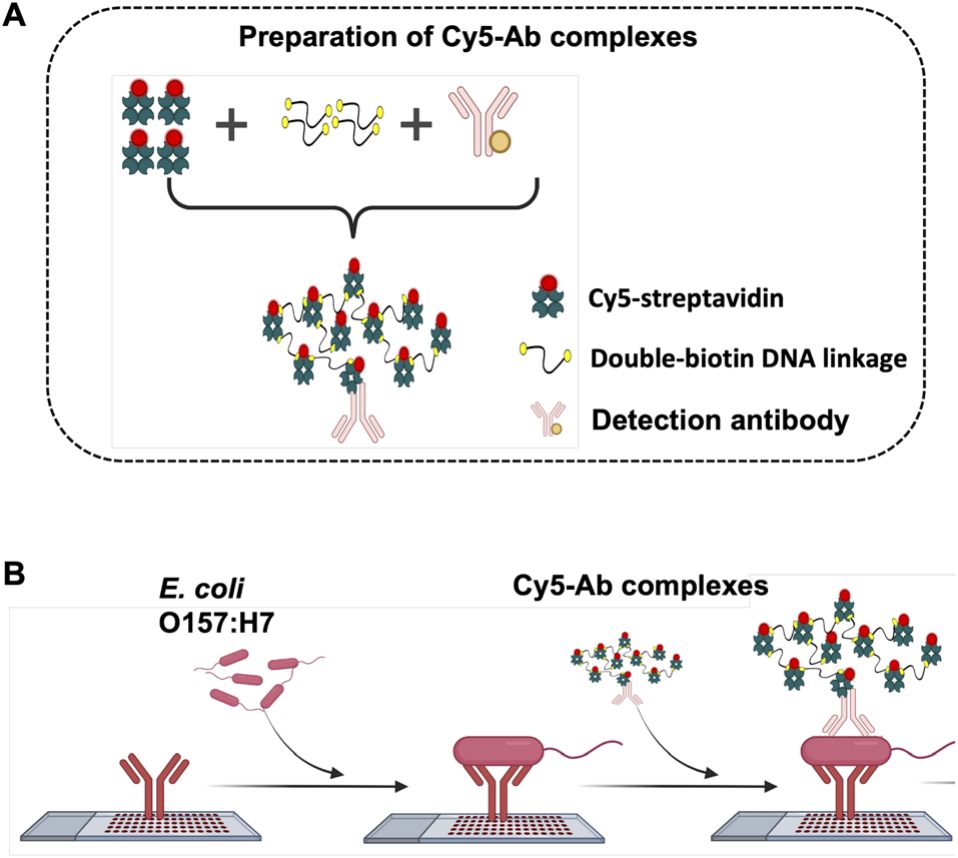
(A) Preparation of Cy5-Ab complexes by attaching Cy5-streptavidin bound double-biotin DNA linkage with detection antibody. (B) The scheme represents the use of Cy5-Ab complexes as a label in the antibody array.

### Detection of foodborne pathogens using a florescent sandwich immunoassay

Aliquots (150 μL) of various 10-fold serial dilutions of heat-killed bacteria in working buffer were added to each array and incubated for 45 minutes at RT. After rinsing with washing buffer 3 times, 150 μL of Cy5-Ab complexes (for monoplex detection) or 300 μL of mixed reporter Cy5-Ab complexes (for duplex detection) was added and incubated for 1 h at RT. After 3× washing, the cassette was removed, and slides were dried with N_2_ gas. The fluorescence signals were read and recorded using Cytation5 imaging reader with GEN5 3.05 imager program at 628 nm emission and 685 nm excitation. The fluorescence image of each spot was then recorded using the Image J program. The average fluorescence intensities of E and S of target, and PC and NC spots were obtained accordingly. The normalized signal of ES-biochip device was computed according to the test value by subtracting the average value of negative control in each array. For stability studies, the Cy5-Ab complexes label and capture antibody of the ES-biochip were examined by keeping at 4 °C for 0–90 days and 0–105 days, respectively. Monoplex *E. coli* O157:H7 with 10^4^ CFU mL^−1^, and duplex *E. coli* O157:H7 and *S. typhimurium* with 10^4^ CFU mL^−1^ were employed to study the stability of Cy5-Ab complexes and ES-biochip every 15 days.

### Real samples detection

The commercial milk and mixed fruit juice UHT were purchased from a supermarket in Bangkok, Thailand. The samples were spiked by heat-killed of *E. coli* O157:H7 and *S. typhimurium,* as desired, using serial dilutions of 1 : 10. Subsequently, 150 μL of the aliquot of the spiked sample was loaded on the ES-biochip array and then followed the detection method.

## Results and discussion

**Fig. 1:**
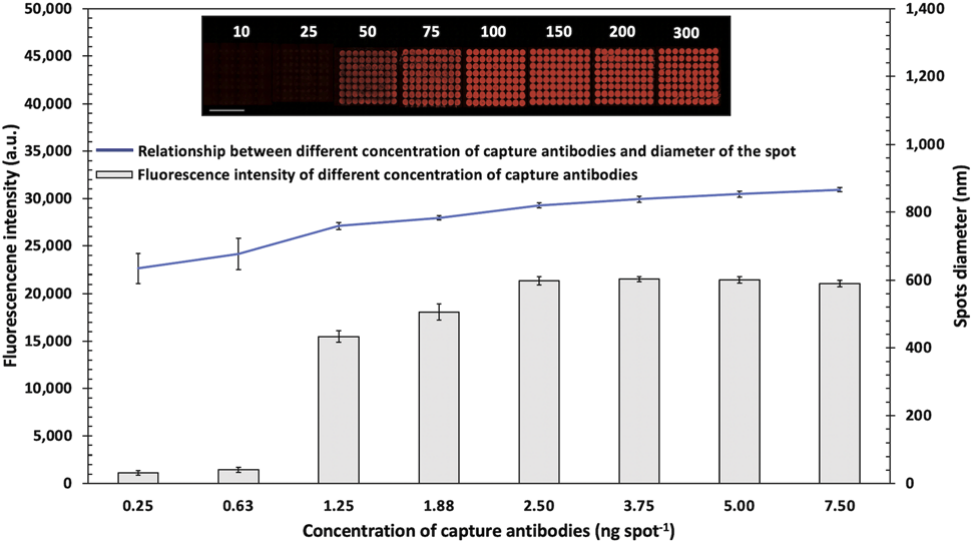
Schematics and fluorescence signals of various concentrations of capture antibodies. (A) Schematic detection of sandwich immunoassay using Cy5-streptavidin as labelling. (B) Fluorescent intensities of antibody arrays at various concentrations of capture antibodies 10–300 μg mL^−1^ with a volume of 25 nL (0.25–7.5 ng per spot) on each microarray spot for detecting 10^6^ CFU mL^−1^ of *E. coli* O157:H7. Error bars show ± 1 std. dev. (*n* = 9). Inset shows Fluorescence images (scale bar = 2000 μm).

To develop a sensitive biosensor, various optimal conditions for the sandwich antibody microarray detection system were performed using the Cy5-streptavidin tagged with a reporter antibody and analyzed by a fluorescence microscope. The concentration of spotting capture antibodies on a glass slide is generally related to the binding efficiency and the size of spotting. The step-by-step schematic fabrication of the microarray with array 9 × 8 = 72 spots for pathogen detection was revealed in Fig. 1A. The bottom ninth row was set as the positive control for the deposited IgG-biotin. Various concentration of capture antibodies (10–300 μg mL^−1^ of anti-*E. coli* O157:H7) were immobilized and analyzed. The fluorescence intensities and diameter of each spot were obtained, as shown in Fig. 1B. The results showed that the concentration of capture antibody (0.25–7.5 ng per spot) provided increased fluorescence intensities with larger diameter of microarray spots. When capture antibody concentration was loaded more than 3.25 ng per spot, the corresponding spots tended to have background smeared in an area of biochip due to the excess antibodies were rinsed off during blocking step. Therefore, to avoid cross-contamination of antibodies array, 150 μg mL^−1^ was chosen as the optimum concentration of capture antibody.

### Fabrication and characterization of Cy5-Ab complexes label

Given an optimal condition of fluorescent molecules tagged with the detection antibody played an important role in identifying the contaminating pathogens. Scheme 1A shows the preparation method of Cy5-Ab complexes. Cy5-streptavidin were attached with the double-biotin DNA linkages to form Cy5-streptavidin/double-biotin DNA. Then biotinylated detection antibody (anti-*E. coli* or anti-*Salmonella*) was exploited to form Cy5-Ab complexes and was used as the label for *E. coli* O157:H7 and *S. typhimurium* detection as shown in Scheme 1B. These three biomolecules were mixed step by step at a 1 : 1 : 1 volume ratio. So, the designed labelling could improve the sensitivity since the huge amounts of Cy5 in a binding event was derived. Cy5-Ab complexes were used as the label for sandwich antibody array with high sensitivity as demonstrated in Fig. S1.† As a result, Cy5-Ab complexes (with double biotin linkage) could amplify the fluorescence signal about twice as compared with a mixed Cy5-streptavidin and antibody (without double biotin linkage).

Additionally, the ability of the detection antibodies to bind to Cy5-streptavidin and double-biotin DNA linkage was taken advantage to provide optimal fluorescent signals. Due to the importance of the concentration of Cy5-streptavidin with double-biotin DNA linkage to get the maximum sensitivity of the fluorescent immunoassays and reduce cell incubation time, we first determined the optimal concentration of Cy5-streptavidin combined double-biotin DNA linkage to maximize the fluorescence intensities.

The optimization conditions for preparation of Cy5-Ab complexes, such as concentration of Cy5-streptavidin and double-biotin DNA linkage were studied to obtain a highly sensitive antibody array and to reduce the measuring steps and minimize time of detection. Concentration of Cy5-streptavidin at 10–80 μg mL^−1^ was performed with 80 fM of double-biotin DNA linkage and 20 μg mL^−1^ of detection antibody for detecting 10^6^ CFU mL^−1^ of *E. coli* O157:H7 as shown in Fig. S2A.† The findings demonstrated that the fluorescent intensities rose when the concentration of Cy5-streptavidin increased up to 60 μg mL^−1^ with minimal non-specific background. Hence, 60 μg mL^−1^ of Cy5-streptavidin were used for the subsequent experiments. Otherwise, the concentration of double-biotin DNA linkage between 4 and 120 fM were optimized and test 10^6^ CFU mL^−1^ of *E. coli* O157:H7 in the array. Fig. S2B† shows that the fluorescent signals increased along with the concentrations of double-biotin. Otherwise, the fluorescence intensities induced by the biochips were lower at 80–120 fM. The possibility of this effect would be that it occupies all four binding sites of streptavidin and does not reduce the binding efficiency of detection antibody. Therefore, 60 fM of double-biotin DNA linkage were chosen for acquiring the highest fluorescent signals.

**Fig. 2:**
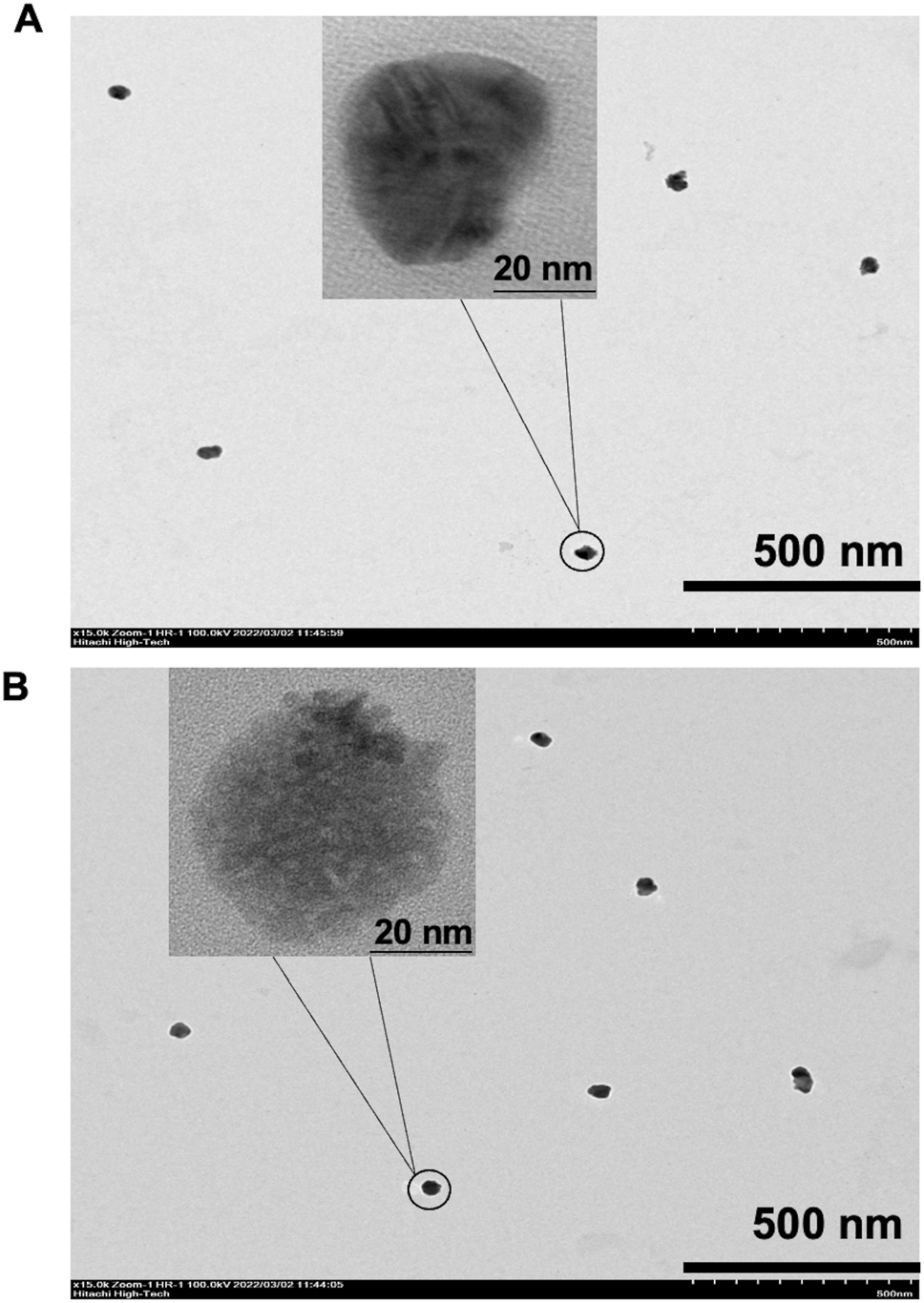
TEM images. (A) Cy5-double biotin-DNA and (B) Cy5-Ab complexes stained by silver.

To characterize the structure of Cy5-double-biotin DNA linkage (Fig. 2A) and Cy5-Ab complexes (Fig. 2B) using silver staining DNA, TEM images were analyzed. The positive charge of Ag^+^ ions bound to the negative charge of phosphodiester backbones of DNA and antibodies to form silver-associated complexes.^22^ Images show the clumping structure like a rock or gravel shape for both. The average area of Cy5-double-biotin DNA clumping was about 64.20 ± 10.83 nm^2^. Otherwise, the size of Cy5-Ab complexes was about 84.67 ± 27.39 nm^2^. The average diameter of Cy5-Ab complexes was bigger than Cy5-double-biotin DNA linkage about 11 nm, which it related to the size of the immobilized antibody (size of IgG is about 5.3 × 10 nm (ref. 23)).

### Optimization conditions for antibody-array

Incubation time of bacteria cells and Cy5-Ab complexes were optimized by detecting 10^3^ CFU mL^−1^ of *E. coli* O157:H7 using the simple array. Fig. S3A† shows that cell binding attained a stationary phase between 45 and 120 min as the fluorescent intensities leveled off. The cell binding time at 45 min was chosen to as a cell incubation time for the pathogen detection. Meanwhile, the fluorescent intensities of different Cy5-Ab complex incubation times are shown in Fig. S3B.† The results show that the intensities were stable (*p* < 0.05) during 45–120 min of incubation time. In particular, 60 min of Cy5-Ab incubation time was selected and used for further experiments as it ensured this time duration was sufficient for optimum binding in a system with both *E. coli* O157:H7 and *S. typhimurium*.

### Design pattern of ES biochip

**Scheme 2:**
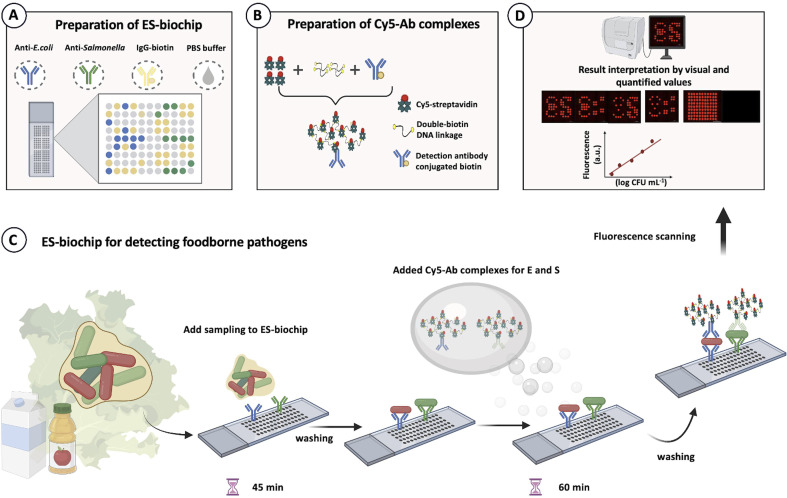
Microarray-patterned biochip design for duplex foodborne pathogen detection. (A) Preparation of *E. coli*–*Salmonella* (ES) biochip. Blue and green microarray spots indicate the locations for capture antibodies (anti-*E. coli* and anti-*Salmonella*, *n* = 10), respectively. The yellow spots show positive control (IgG-biotin, *n* = 28) and grey spots represent negative (1× PBS). (B) Preparation of Cy5-Ab complexes to amplify a foodborne detection signal. (C) ES-biochip for detecting foodborne pathogens. (D) Fluorescence image analysis.

The biochip seen with the ES symbols was designed for duplex pathogen detection of *E. coli* (E symbol) and *Salmonella* (S symbol) for easier reading of measured results and observation. It was designed in such a way that each microarray spot depicted different pathogen species with positive and negative controls. Scheme 2A shows that the blue and green microarray dots represented the capture antibodies for anti-*E. coli* O157:H7 and anti-*Salmonella*, respectively. While the yellow dots corresponded to IgG-biotin antibodies acting as the immobilized antibodies on the SuperFrost® Plus slide, serving as a positive control (PC). On the other hand, 1× PBS was used as the negative control (NC), showing with the grey dots. The center-to-center spacing between the microarray spots was set to be 1 mm. The microarray-based biochip could detect both species with the “ES” notation.

### Cross-reactivity and selectivity

A brief schematic for monoplex and duplex detection using ES-biochip is shown in Scheme 2C and D. When *E. coli* O157:H7 was found on the microarray assay, the symbol “E” appeared. Similarly, the symbol “S” indicated that *S. typhimurium* had been detected in the assay. To assure the fluorescent signal obtained by Cy5-streptavidin for the negative control, none of the microbes was deposited on the assay and its corresponding control signal was observed for the biochip. With this pattern design for the appearance of the ES symbols, the microarray-based biochip potentially allowed the effective visual determination for the duplex pathogen detection.

**Fig. 3:**
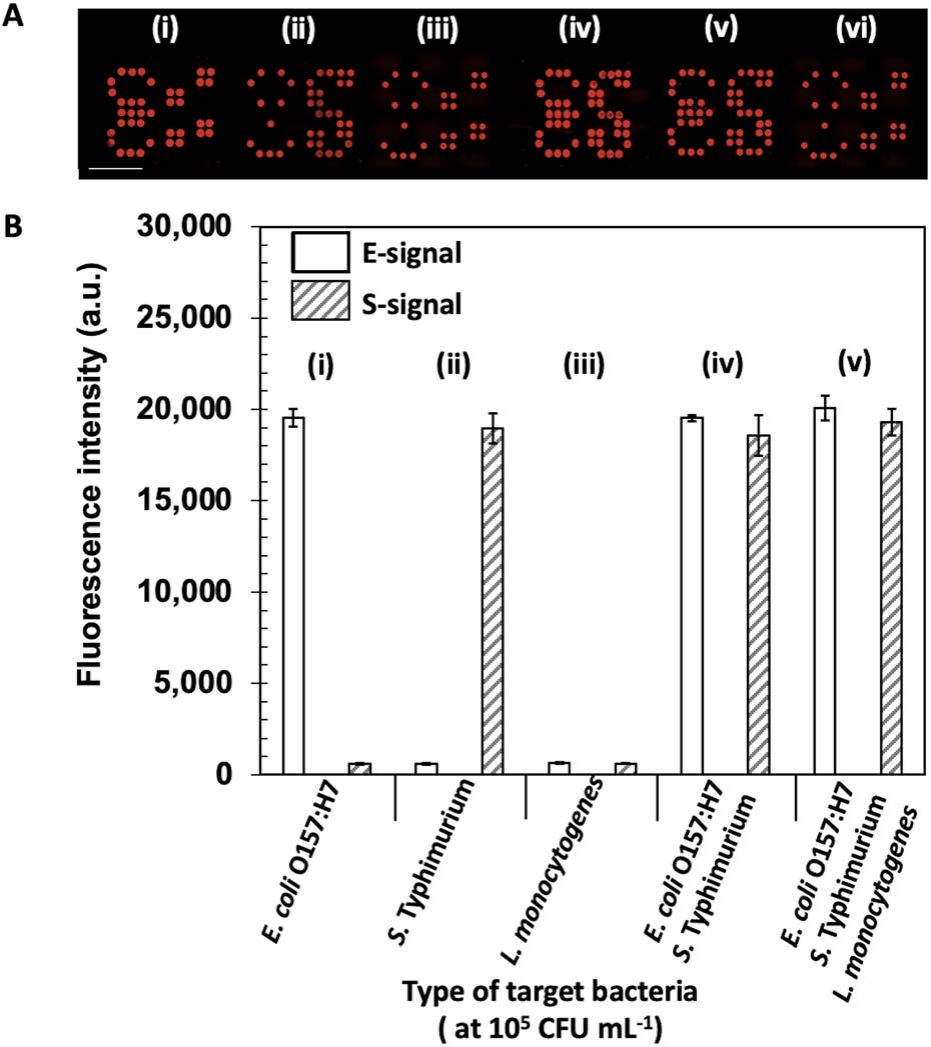
Selectivity test. (A) Fluorescent images of the biochips show the cross-reactivity tests by depositing (i) only *E. coli* O157:H7, (ii) *S. typhimurium*, (iii) *L. monocytogenes*, (iv) *E. coli* O157:H7 and *S. typhimurium*, (v) *E. coli* O157:H7, *S. typhimurium*, and *L. monocytogenes* of each cell concentration of 10^5^ CFU mL^−1^, (vi) without bacteria cell (B) Fluorescence intensities were obtained for each pathogen deposited for the selectivity tests. Error bars show ± 1 std. dev. (*n* = 10).

The cross-reactivity and selectivity were carried out toward the loading 10^5^ CFU mL^−1^ of *L. monocytogenes*, *E. coli* O157:H7, and *S. typhimurium* onto the ES-biochip. First, *E. coli* O157:H7 was deposited on the patterned assay as shown in Fig. 3A(i). The ES-biochip was able to obtain the only “E” symbol with its positive control sign. Thereby, the selectivity of the patterned assay was achieved through the injection of monoplex *E. coli* O157:H7 as revealed by Fig. 3B(i). While the injection of monoplex *S. typhimurium* on the assay was shown in Fig. 3A(ii), the selectivity was determined in Fig. 3B(ii). In this case, the symbol “S” was successfully observed on the biochip with its corresponding positive control sign. The biochips for monoplex detection ascertained that the patterned microarray assays efficiently maintained high selectivity toward the microbe attachment. With only non-target cells of *L. monocytogenes* added onto the assay as exhibited on Fig. 3A(iii), neither fluorescent “E” nor “S” symbols appeared on it and very minimal fluorescence signal was measured by the assay as displayed by Fig. 3B(iii). In this condition, two positives control signs were seen to confirm the biochip performed properly. While incubating both microbial cell lines of *E. coli* O157:H7 and *S. typhimurium* as illustrated in Fig. 3A(iv), the resultant symbols of “E” and “S” were shown simultaneously on the microarray assay with their fluorescence intensities represented in Fig. 3B(iv). Similarly for the case as all three microbial cell lines injected on the biochip shown in Fig. 3A(v), it still efficiently detected both *E. coli* O157:H7 and *S. typhimurium* visually with well-performance demonstrated by Fig. 3B(v) whereas Fig. 3A(vi) does not express symbols of “E” and “S” in the absence of bacterial cells.

To assure the assay working accurately, the two positive control signs as only the detection antibodies attached with Cy5-streptavidin was calibrated and shown in Fig. 3A(vi). The fluorescence signal of the positive and negative control and *L. monocytogenes* nontarget bacteria was significantly different from the *E. coli* O157:H7 and *S. typhimurium* target bacteria, which indicated that the platform detection was highly specific.

### Characterization of ES-Biochip on monoplex and duplex detection

**Fig. 4:**
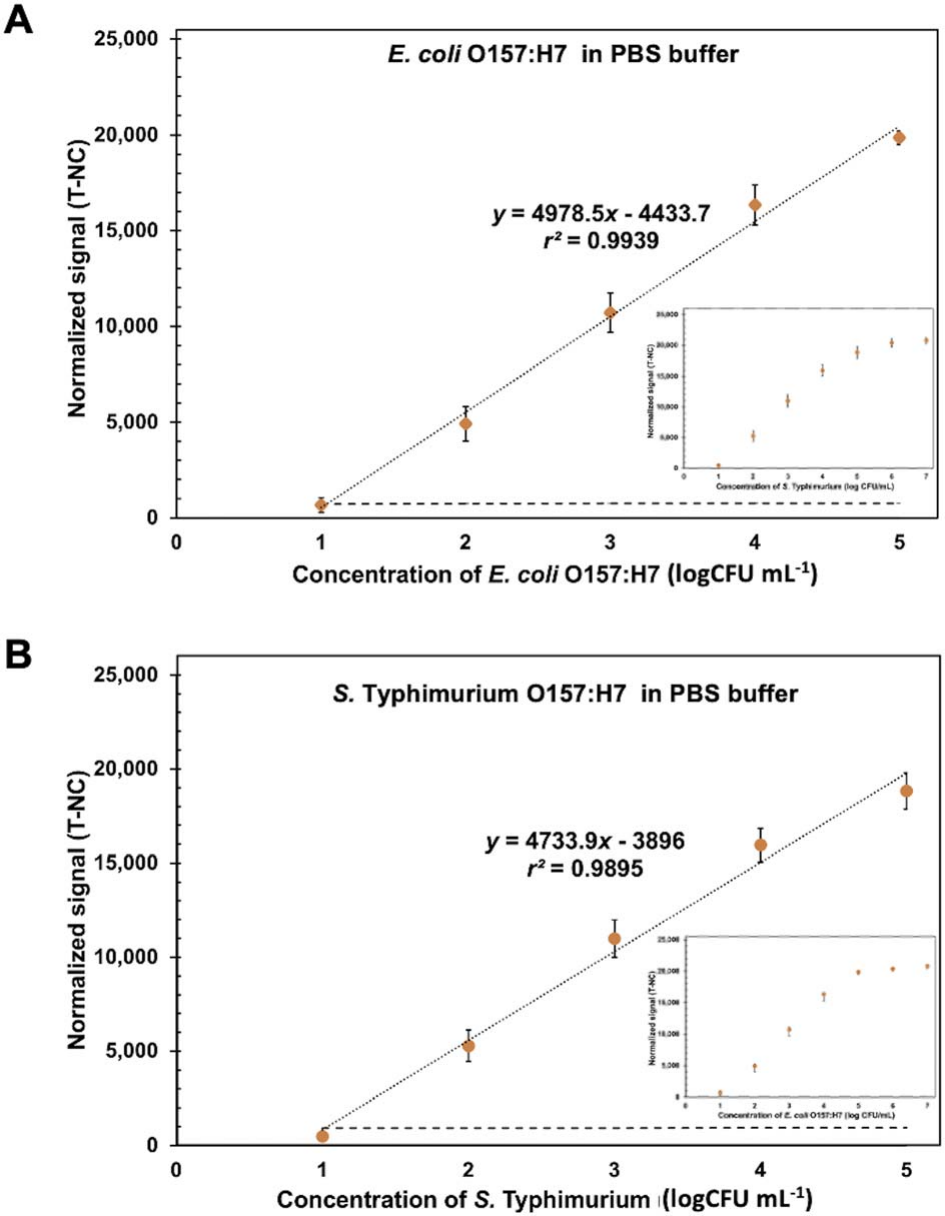
Calibration curve for *E. coli* O157:H7 and *S. typhimurium* detection. The linear calibration curves between the normalized signal (T-NC) and the logarithm concentration of (A) *E. coli* O157:H7 and (B) *S. typhimurium* from 10 to 10^5^ CFU mL^−1^ in 1× PBS using the ES-biochip with Cy5-Ab complexes label. Error bars show ± 1 std. dev. (*n* = 10). Inset show the calibration curve between 10 and 10^7^ CFU mL^−1^ of bacteria.

Under the optimization conditions, the ES-biochips coupling Cy5-Ab complexes labels (scheme shown in Fig. S4A†) were tested with different loading concentrations from 10 to 10^5^ CFU mL^−1^ of *E. coli* O157:H7 and *S. typhimurium* in 1× PBS. The visual observation from 10^3^ to 10^7^ CFU mL^−1^, resulted in Fig. S4B.† The normalized fluorescent intensity (signal of each spot for E or S was subtracted by average signal of NC in each array; T-NC) was plotted against the concentration of pathogenic cells derived from mono and duplex detection. The results showed that the linearity range for detecting *E. coli* and *S. typhimurium* is between 10 and 10^5^ CFU mL^−1^. Additionally, the high corelation (99.95%) between monoplex and duplex detection are presented, as shown in Fig. S4C and D.† This implied that the assay allowed the parallel and independent detection for both microbial species as the detection of each species did not interfere with one another. Therefore, the average linear relationships between the normalized intensity and concentration of *E. coli* O157:H7 ((T-NC) = 4978.5 × (log *c* [CFU mL^−1^]) − 4433.7) and *S. typhimurium* ((T-NC) = 4733.9 × (log *c* [CFU mL^−1^]) − 3896) were performed, as shown in Fig. 4. The limit of detection (LOD) was calculated based on *x*′ + 3*s,* where *x'* is the mean signal of cell-free buffer and *s* is the standard deviation of that one. LODs of *E. coli* O157:H7 and *S. typhimurium* were found to be 9 CFU mL^−1^ and 7.83 CFU mL^−1^, respectively. Although this ES-biochip conjugated Cy5-Ab complexes does not exhibit the wide linear range, lowest LOD for *E. coli* and *S. typhimurium* in antibody arrays was obtained (Table S1†). Also, the Cy5-Ab complexes allows for amplification signal reducing the detection step.

### Stabilities of ES-biochip and Cy5-Ab complexes

**Fig. 5:**
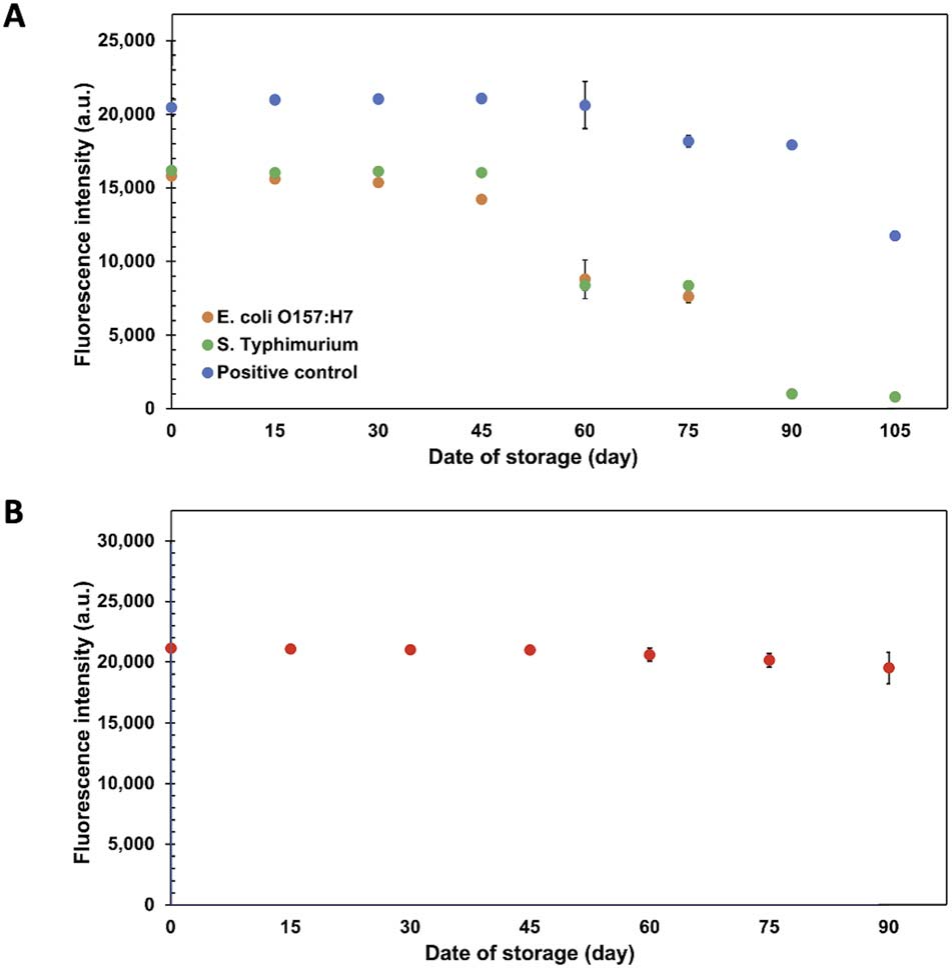
Stabilities of ES-biochip and Cy5-Ab complexes. (A) Fluorescence intensities of positive control and 10^4^ CFU mL^−1^ of *E. coli* O157:H7 and *S. typhimurium* using ES-biochip, stored at 4 °C, with Cy5-Ab complexes as label. (B) Fluorescence intensities of 10^4^ CFU mL^−1^ of *E. coli* O157:H7 using simple array with Cy5-Ab complexes stored at 4 °C. Error bars show ± 1 std. dev. (*n* = 10).

To assure point-of-need food safety screening, the microarray biochip is needed to attain high-storage performance with reliable stability. In this study, the long-term storage of the Cy5-Ab complexes labels and ES-biochip was examined by keeping at 4 °C. The biochips were taken for duplex detection of 10^4^ CFU mL^−1^ of *E. coli* O157:H7 and *S. typhimurium* for every 15 days, the results show in Fig. 5A and S5.† The measured fluorescence intensities were relatively invariant for *E. coli* O157:H7, *S. typhimurium* and positive control. The storage of ES-biochip decreased after 45 days. There was a slightly decrease in the efficiency of positive control. Cy5-Ab complexes were tested with 10^4^ CFU mL^−1^ of *E. coli* O157:H7 on simple array. Fig. 5B shows that the Cy5-Ab complexes can storage more than 90 days.

The reproducibility of the assay was estimated from the stability test which performed the repeated measurements on a different day, derived from the stability test. The intra-day relative standard deviation (RSD) was 0.3–14.8% (*n* = 10) and the inter-day RSD was 4.67% across 45 days (*n* = 4).

### Duplex pathogen detection in food samples

**Fig. 6:**
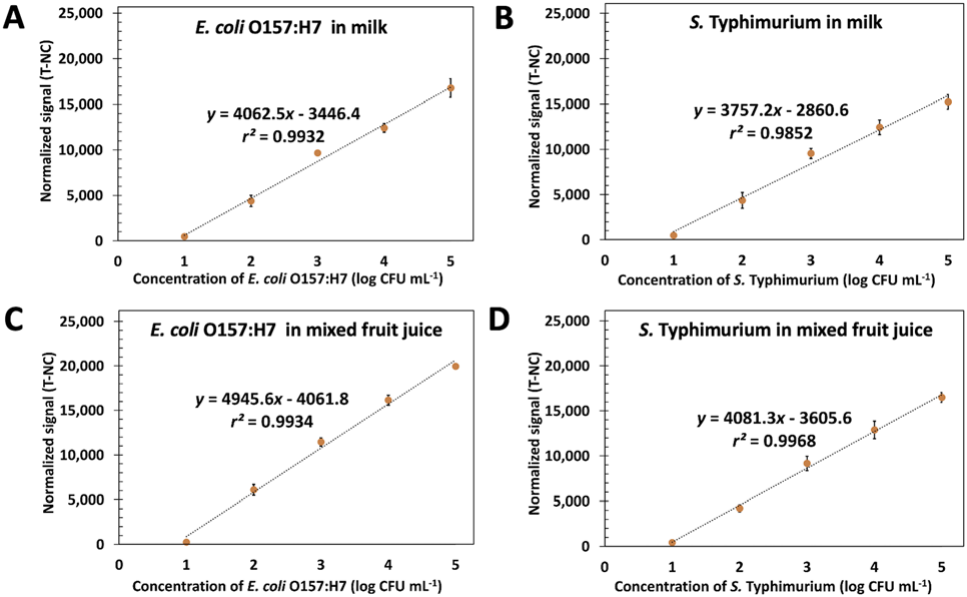
Linear calibration curves in milk and juice. The plots between the normalized signal (T-NC) and the logarithm concentration of *E. coli* O157:H7 and *S. typhimurium* ranging from 10 to 10^5^ CFU mL^−1^ in milk (A and B) and mixed fruit juice (C and D) using ES-biochip with Cy5-Ab complexes.

Food matrix, especially protein and fat, usually reduces the signal in the biosensor system. Here the spiked heat-killed 10–10^5^ CFU mL^−1^ of *E. coli* O157:H7 and *S. typhimurium* in milk and fruit juice UHT were tested by using ES-biochip with Cy5-Ab complexes and the results of duplex detection are demonstrated in Fig. 6A–D. The normalized fluorescent signals and logarithm concentration of each pathogen correlated linearly. The visual detection for both spiked pathogens was still observed on the ES-biochip assay (Fig. S6A and B†). For monoplex and duplex detection in milk and juice samples, the results illustrated that two detection methods produced similar results, as shown in Fig. S6C and D.† By the way, the matrix of milk had the effect of reducing the sensitivity by 18.87%. The LODs of 8.4 and 7.2 CFU mL^−1^ of *E. coli* O157:H7 in milk and juice, respectively, were calculated by using linear graphs in Fig. 4A. *S. typhimurium* spiked in milk and juice gave the LOD (calculated by using linear graph in Fig. 4B) of 7.2 and 8.5 CFU mL^−1^, respectively. Table S2† show %recovery of *E. coli* O157:H7 and *S. typhimurium* spiked in milk and juice was 83–109% and 81–96%, respectively. The Cy5-Ab complexes conjugated ES-biochip platform has potential for foodborne detection in industry to reduce labor and assay time. By exploiting the detection tests on the real samples, these results revealed that Cy5-Ab complexes hold great potential for the sensitive detection of other pathogens as well.

## Conclusions

We developed an ES-biochip to be used with Cy5-Ab complexes label for simultaneously detection of foodborne pathogens. The ES-biochip allowed for the visual and quantified detection of foodborne bacteria. Furthermore, the long-term stable of Cy5-Ab complexes are successfully preparation and using in the antibody-arrays by reducing the detection steps and time. High selectivity and specificity for detecting these pathogens in buffer and commercial drinks, such as milk and mixed fruit juice UHT, were performed.

## Author contributions

T. H. carried out the experiments and wrote the manuscript with guidance from P. R., and S. K. and W. S. P. R. and T. H. designed pattern for the ES-biochip. P. R. conceived the original idea. S. K., P. R. and W. S. supervised the project, edited, and reviewed the manuscript.

## Conflicts of interest

The authors declare no competing financial interests regarding the publication of this paper.

## Supplementary Material

RA-012-D2RA03391G-s001
