# Supplementary material for: Highly sensitive and selective antibody microarrays based on a Cy5-antibody complexes coupling ES-biochip for E. coli and Salmonella detection
Source: RSC Adv. 2022 Aug 31;12(38):24760–8. doi: 10.1039/d2ra03391g (PMC9429895; doi:10.1039/d2ra03391g)
Supplement: RA-012-D2RA03391G-s001 [file RA-012-D2RA03391G-s001.pdf]

Electronic Supplementary Material (ESI) for RSC Advances.
This journal is © The Royal Society of Chemistry 2022

Supplementary Information

Highly sensitive and selective antibody microarrays based on Cy5-Antibody complexes coupling ES-biochip for *E. coli* and *Salmonella* detection

Timpika Homsombut[a], Patsamon Rijiravanich[b]*, Werasak Surareungchai[a,c], Surachate Kalasin[a**]

[a] *Faculty of Science and Nanoscience & Nanotechnology Graduate Program, King Mongkut's University of Technology Thonburi, 10140, Thailand*
[b] *BioSciences and Systems Biology Research Team, National Center for Genetic Engineering and Biotechnology, National Sciences and Technology Development Agency at King Mongkut's University of Technology Thonburi, 10150, Thailand*
[c] *School of Bioresources and Technology, King Mongkut's University of Technology Thonburi, 10150, Thailand*

*Corresponding authors Email: *patsamon.rij@biotec.or.th, **surachate.kal@kmutt.ac.th
ORCID: 0000-0002-7080-9513 (Rijiravanich, P.)
ORCID: 0000-0001-6413-6642 (Kalasin, S.)

Content

1. Comparison of the fluorescent signal between Cy5-streptavidin and Cy5-Ab complexes.
2. Optimization conditions for preparation of Cy5-Ab complexes.
3. Optimization conditions for detection
4. Mono- and Duplex detection in buffer on ES-biochips.
5. Stability test for ES-biochip and Cy5-Ab complexes.
6. Evaluation of ES-biochip by food samples.
7. **Table. S1** Comparison of the overall analytical performance using antibody array for *E. coli* and *Salmonella* detection.
8. **Table. S2** The recovery of *E. coli* O157:H7 and *S.* Typhimurium detection in food samples testing.

Comparison of the fluorescent signal between Cy5-streptavidin and Cy5-Ab complexes.

Experiment

The flow chart below shows the step by step of measuring *E. coli* by using Cy5-streptavidin and Cy5-Ab complexes.

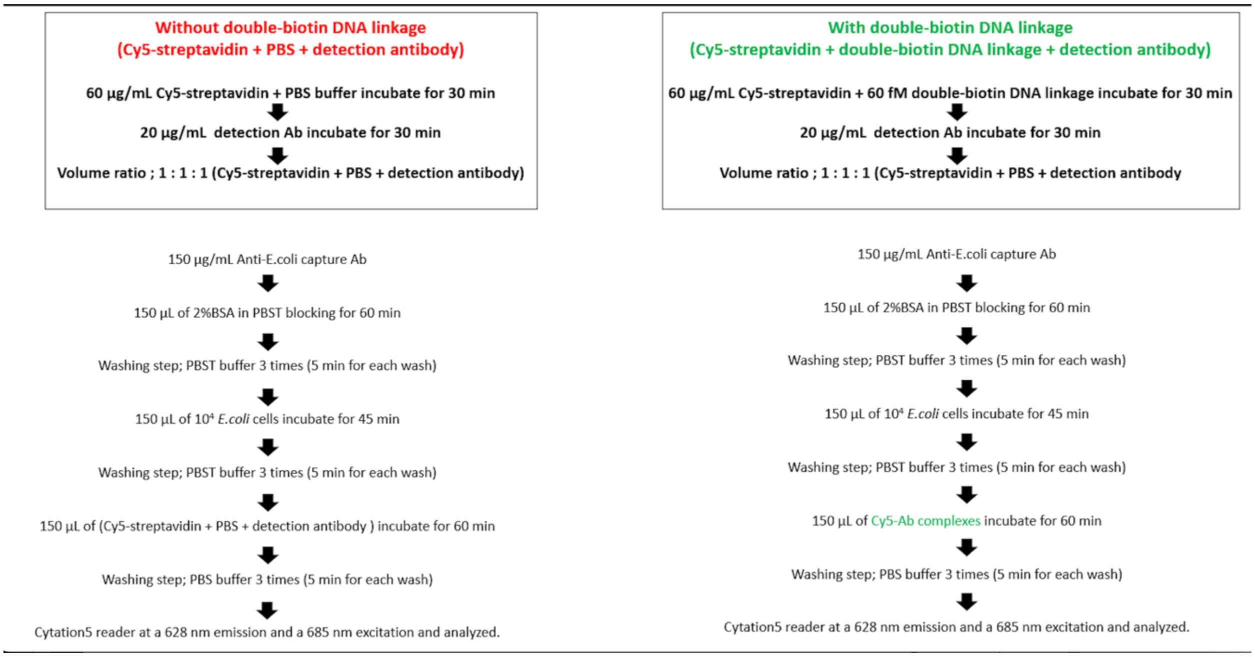

Result

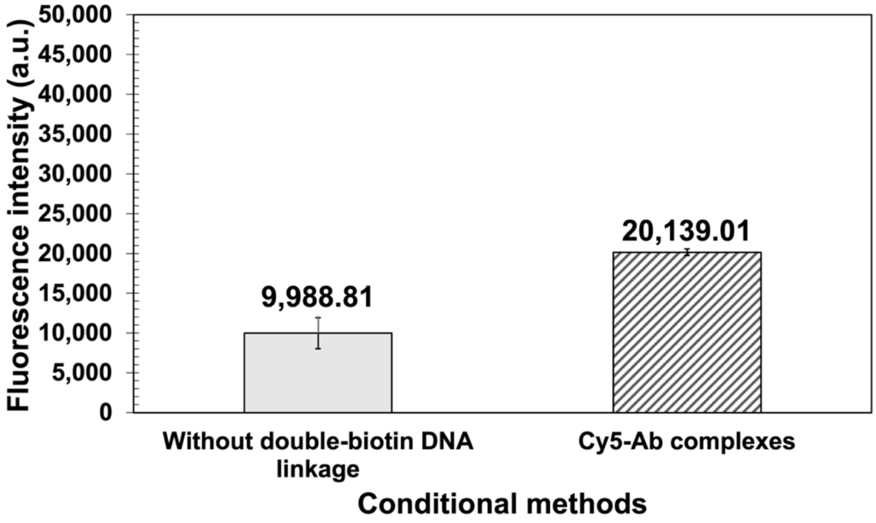

Fig. S1 Comparison of the fluorescent signal between Cy5-streptavidin and Cy5-Ab complexes as label for detection of 10^3 CFU/mL of *E. coli* O157:H7 using simple array. Error bars show ±1 std. dev. (n = 9).

Optimization conditions for preparation of Cy5-Ab complexes

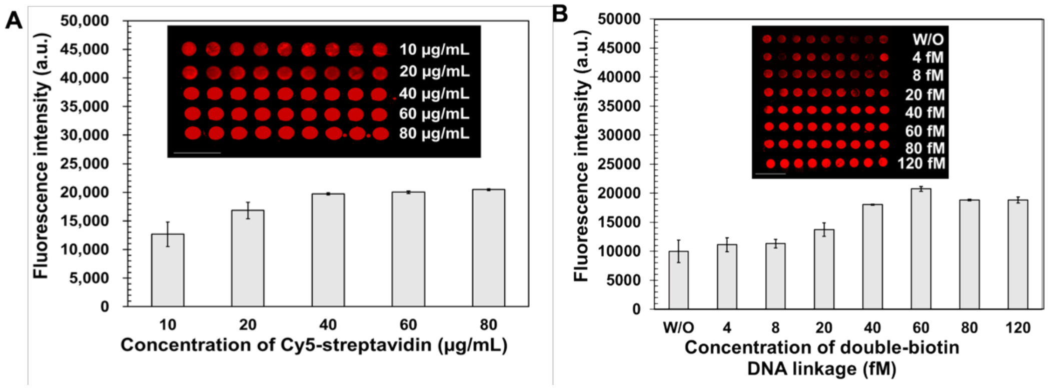

Fig. S2 Optimization conditions for preparation of Cy5-Ab complexes. Fluorescent intensities and images (inset) of 10^4 CFU/mL of *E. coli* O157:H7 using simple array at various concentrations of (A) Cy5-streptavidin and (B) double-biotin DNA linkage. Error bars show ±1 std. dev. (n = 9).

Optimization conditions for detection

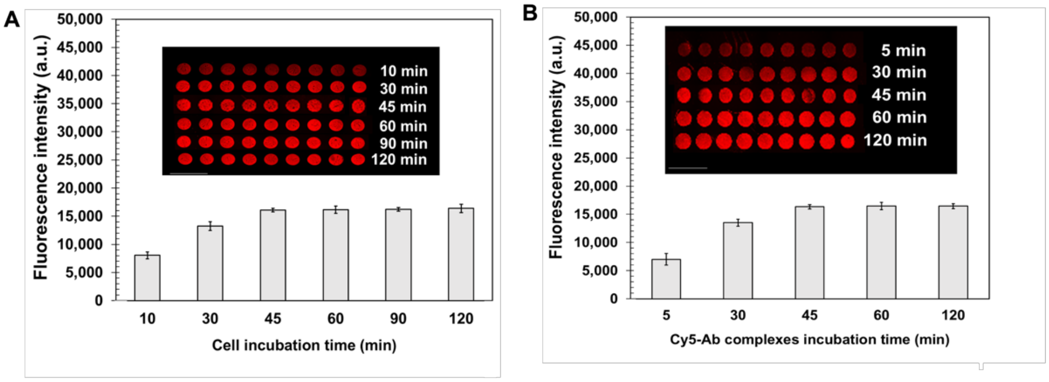

Fig. S3 Optimization conditions for incubation time of (A) cell bacteria and (B) Cy5-Ab complexes on antibody array. Fluorescent intensities and image (inset) of 10^4 CFU/mL of *E. coli* O157:H7 using simple array at various incubation time of (A) target bacteria and (B) Cy5-Ab complexes. Error bars show ±1 std. dev. (*n* = 9).

Mono- and Duplex detection in buffer on ES-biochips

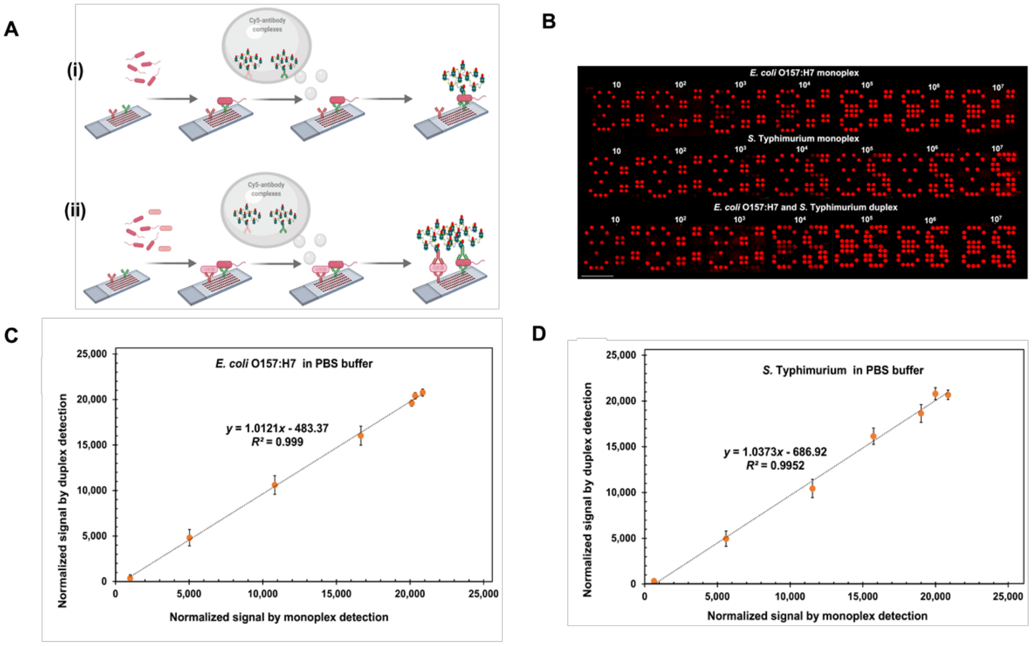

Fig. S4 Monoplex and Duplex detection in buffer on ES-biochips. (A) Schematic detection of (i) monoplex and (ii) duplex using ES-biochip conjugated Cy5-Ab complexes label. (B) The fluorescence images for duplex detection of 10-10^7 CFU/mL of *E. coli* O157:H7 and *S.* Typhimurium. Comparison between monoplex and duplex detection by (C) *E. coli* O157:H7 and (D) *S.* Typhimurium detection in PBS.

Stability test for ES-biochip and Cy5-Ab complexes

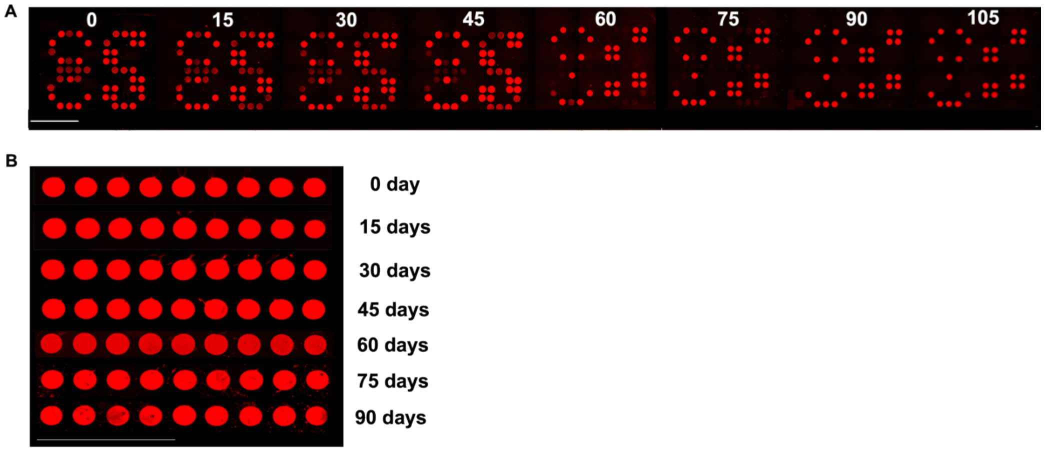

Fig. S5 Stability test for (A) ES-biochip and (B) Cy5-Ab complexes. Image of fluorescence intensities of (A) ES-biochip, stored at 4 °C, detected 10^4 CFU/mL of *E. coli* O157:H7 and *S.* Typhimurium with Cy5-Ab complexes as label. (B) Image of fluorescence intensities of 10^4 CFU/mL of *E. coli* O157:H7 using simple array with Cy5-Ab complexes stored at 4 °C.

Evaluation of ES-biochip using food samples

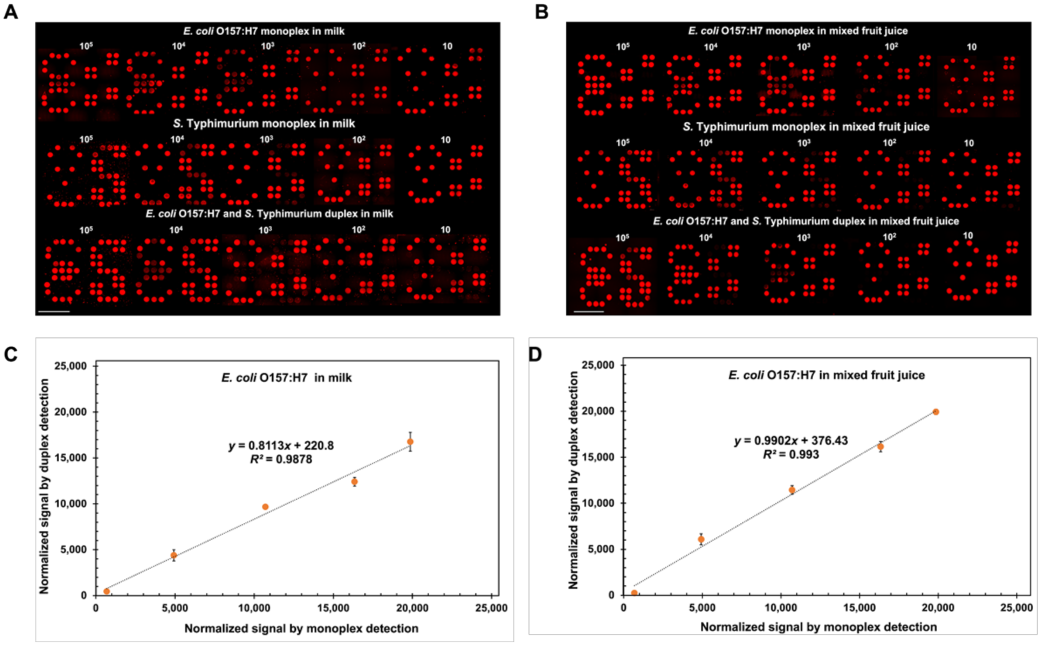

Fig. S6 Real sample tests*.* The fluorescence images for the duplex detection of different concentrations of spiked *E. coli* O157:H7 and *S.* Typhimurium ranging from 10 to 10^5 CFU/mL in (A) commercial milk and (B) mixed fruit juice using the ES-biochips. Comparison signals of *E. coli* O157:H7 detection in (C) PBS and milk and (D) PBS and juice.

Table. S1 Comparison of the overall analytical performance using antibody array for *E. coli* and *Salmonella* detection.

Pathogens	Label	Detection limit (CFU/mL)	Detection range	Assay time (h)	Samples	Ref.
E. coli O157:H7	Horseradish peroxidase (HRP)	8×10^4	2×10^4 - 5×10^7	1.15	Milk	1
Salmonella spp.		5×10^7	1×10^6 - 1×10^9			
E. coli O157:H7	Cy3	1×10^6	1×10^6 - 1×10^9	2.5	-	2
Salmonella spp.		1×10^7	1×10^7 - 1×10^9			
E. coli O157:H7	Horseradish peroxidase (HRP)	1.5×10^6	1×10^5 - 1×10^6	1	Milk	3
Salmonella spp.		1.3×10^7	1×10^6 - 1×10^7			
Salmonella spp.	Label-free	7.6×10^6	5.14×10^4 – 5.14×10^8	1.30	Chicken rinse matrix	4
E. coli O157:H7	Alexa Fluor 555	5.8×10^5	6.4×10^4 – 1.4×10^8	1.15	-	5
Shiga toxin 1 (Stx1)		110 ng/mL	1.4×10^0 – 9×10^3 ng/mL			
E. coli O157:H7	Alexa-Fluor 647	1×10^3	1×10^1 - 1×10^9	2	Beef, chicken, and turkey	6
Salmonella spp.		1×10^3	1×10^1 - 1×10^9			
L. monocytogenes		1×10^3	1×10^1 - 1×10^9			
E. coli O157:H7	Cy5-Ab complexes (Signal amplification)	8.4	1×10^1 - 1×10^5	1.45	Milk	This work
S. Typhimurium		7.2				
E. coli O157:H7		7.2			Mixed fruit juice	
S. Typhimurium		8.5				

Table. S2 The recovery of *E. coli* O157:H7 and *S.* Typhimurium detection in food samples testing.

Samples	Pathogens	Spiked concentration (CFU/mL)	Detected concentration (CFU/mL)	Recovery (%)
**Milk**	*E. coli* O157:H7	1	0.96	98.4
		5	4.259	85.2
	S. Typhimurium	1	0.89	89.02
		5	4.81	96.3
**Mixed fruit juice**	*E. coli* O157:H7	1	0.94	94.04
		5	4.89	97.9
	S. Typhimurium	1	0.99	98.7
		5	4.92	98.5

Supplementary References

1. R. Charlermroj, O. Gajanandana, C. Barnett, K. Kirtikara and N. Karoonuthaisiri, *Anal. Lett.*, 2011, **44**, 1085-1099, DOI: 10.1080/00032719.2010.511736.
2. A. G. Gehring, D. M. Albin, S. A. Reed, S.I. Tu and J. D. Brewster, *Anal. Bioanal. Chem*, 2008, **391**, 497-506, DOI: 10.1007/s00216-008-2044-6.
3. N. Karoonuthaisiri, R. Charlermroj, U. Uawisetwathana, P. Luxananil, K. Kirtikara and O. Gajanandana, *Biosens. Bioelectron*, 2009, **24**, 1641-1648, DOI: 10.1016/j.bios.2008.08.026.
4. J. Chen and B. Park, *Anal Bioanal Chem*, 2018, **410**, 5455-5464, DOI: 10.1007/s00216-017-0810-z.
5. A. G. Gehring, J. D. Brewster, Y. He, P. L. Irwin, G. C. Paoli, T. Simons, S.I. Tu and J. Uknalis, *Sensors*, 2015, **15**, 30429-30442, DOI: 10.3390/s151229807.
6. S.H. Ohk and A. K. Bhunia, *Food Microbiology*, 2013, **33**, 166-171, DOI: 10.1016/j.fm.2012.09.013.
